# Potential Explanation of the Reported Association between Maternal Smoking and Autism

**DOI:** 10.1289/ehp.1206268

**Published:** 2013-02-01

**Authors:** William H. James

**Affiliations:** The Galton Laboratory, Department of Genetics, Evolution and Environment, University College London, London, United Kingdom, E-mail: whjames@waitrose.com

Kalkbrenner et al. (2012) reported a positive association between maternal smoking and higher functioning autism syndrome disorder (ASD) subtypes. Other studies had previously reported associations between maternal smoking and ASD (St. Pourcain et al. 2011) and between maternal smoking and attention deficit hyperactivity disorder (Langley et al. 2012). Kalkbrenner et al. (2012) concluded that the association warrants further research. No explanation for an association between autism and maternal smoking has been established, so I would like to suggest one.

Baron-Cohen (2002) proposed that one cause of autism is exposure to high levels of intrauterine testosterone; I recently noted that if the mother is the source of the testosterone, many of the established risk factors for autism may be interpreted as confirmation for this hypothesis (James 2012). Stress causes women to secrete adrenal androgens including testosterone; thus, any maternal-stress–related risk factor for autism may be interpreted as supporting Baron-Cohen’s hypothesis (Baron-Cohen 2002). Some of the many risk factors are well established, having been identified by Gardener et al. (2009) in a comprehensive meta-analysis, for example, advanced parental age at birth, maternal use of prenatal medications, maternal bleeding, gestational diabetes, being firstborn, and having a mother who was born abroad. I have identified other risk factors using a less rigorous criterion, namely, risk factors that had been reported as statistically significant more than once (James 2012). These included low birth weight, short duration of gestation, maternal obesity, mother in a technical occupation, lack of breastfeeding, race, and increasing time trend. No previous explanation had been offered for all of these risk factors collectively.

Evidence suggests that the risk factor reported by Kalkbrenner et al. (2012)—maternal smoking—may increase maternal testosterone levels. For example, androgen levels are reportedly high in women who smoke (Kaergaard et al. 2000; Pölkki and Rantala 2009; Sowers et al. 2001), and this has been specifically reported in pregnant women (Toriola et al. 2011). One may infer that maternal smoking (like the other risk factors for autism cited above) is positively associated with increased levels of intrauterine testosterone and thus—invoking the hypothesis of Baron-Cohen (2002)—with autism.

Beaudet (2012) has recently proposed a category of autism that he designated as a milder, more homogeneous form in which the mean IQ is higher. This category is more subject to the recent reported increase in autism and to environmental, as contrasted with genetic, factors. The data of Kalkbrenner et al. (2012) suggest that the association of autism with maternal smoking is confined to higher-functioning autism subtypes. Thus, I suggest that their data could be explained by the high levels of testosterone associated with maternal smoking.
